# Genetic network analysis of human immunodeficiency virus sexual transmission in rural Southwest China after the expansion of antiretroviral therapy: A population-based study

**DOI:** 10.3389/fmicb.2022.962477

**Published:** 2022-08-18

**Authors:** Jin Chen, Huanhuan Chen, Jianjun Li, Liuhong Luo, Ruihua Kang, Shujia Liang, Qiuying Zhu, Huaxiang Lu, Jinhui Zhu, Zhiyong Shen, Yi Feng, Lingjie Liao, Hui Xing, Yiming Shao, Yuhua Ruan, Guanghua Lan

**Affiliations:** ^1^State Key Laboratory of Infectious Disease Prevention and Control, National Center for Acquired Immune Deficiency Syndrome (AIDS)/Sexually Transmitted Disease (STD) Control and Prevention, Chinese Center for Disease Control and Prevention, Collaborative Innovation Center for Diagnosis and Treatment of Infectious Diseases, Beijing, China; ^2^Guangxi Key Laboratory of Major Infectious Disease Prevention Control and Biosafety Emergency Response, Guangxi Center for Disease Control and Prevention, Nanning, China

**Keywords:** HIV, genetic network, antiretroviral therapy, transmission, prevention

## Abstract

**Background:**

This study is used to analyze the genetic network of HIV sexual transmission in rural areas of Southwest China after expanding antiretroviral therapy (ART) and to investigate the factors associated with HIV sexual transmission through the genetic network.

**Materials and methods:**

This was a longitudinal genetic network study in Guangxi, China. The baseline survey and follow-up study were conducted among patients with HIV in 2015, and among those newly diagnosed from 2016 to 2018, respectively. A generalized estimating equation model was employed to explore the factors associated with HIV transmission through the genetic linkage between newly diagnosed patients with HIV (2016–2018) and those at baseline (2015–2017), respectively.

**Results:**

Of 3,259 identified HIV patient sequences, 2,714 patients were at baseline, and 545 were newly diagnosed patients with HIV at follow-up. A total of 8,691 baseline objectives were observed by repeated measurement analysis. The prevention efficacy in HIV transmission for treated HIV patients was 33% [adjusted odds ratio (AOR): 0.67, 95% confidence interval (CI): 0.48–0.93]. Stratified analyses indicated the prevention efficacy in HIV transmission for treated HIV patients with a viral load (VL) of <50 copies/ml and those treated for 4 years with a VL of <50 copies/ml to be 41 [AOR: 0.59, 95% CI: 0.43–0.82] and 65% [AOR: 0.35, 95% CI: 0.24–0.50], respectively. No significant reduction in HIV transmission occurred among treated HIV patients with VL missing or treated HIV patients on dropout. Some factors were associated with HIV transmission, including over 50 years old, men, Zhuang and other nationalities, with less than secondary schooling, working as a farmer, and heterosexual transmission.

**Conclusion:**

This study reveals the role of ART in reducing HIV transmission, and those older male farmers with less than secondary schooling are at high risk of HIV infection at a population level. Improvements to ART efficacy for patients with HIV and precision intervention on high-risk individuals during the expansion of ART are urgently required.

## Introduction

The aim of the Acquired Immune Deficiency Syndrome (AIDS) prevention and control strategy 95-95-95 is to help end the AIDS epidemic as a public health threat by 2030 (UNAIDS, 2015; Frank et al., 2019). The first “95” of the Joint United Nations Programme on HIV/AIDS (UNAIDS) 95-95-95 goal is 95% of all people living with HIV will know their HIV status, the second “95” is 95% of people with diagnosed HIV infection will receive sustained antiretroviral therapy (ART), and the final “95” is 95% of all people receiving ART will have viral suppression. ART is among the main means of achieving this goal (Lima et al., 2017; Saag et al., 2020). With the continuous improvement of ART strategies, China’s ART standards were adjusted in 2016 to recommend free ART to all HIV-infected persons regardless of their CD4 cell counts, further expanding the scope of treatment. ART has been reported to effectively reduce the viral load (VL) and decline the mortality rate of HIV-infected people (Trickey et al., 2017; Mangal et al., 2019; Zhou et al., 2020). Between 2011 and 2016, the mortality rate of HIV-infected individuals in Western Kenya dropped significantly during the ART scale-up (Otieno et al., 2019). Immediate ART among HIV patients with CD4 cell counts exceeding 500 cells/μl was associated with a 63% reduction in overall mortality (Zhao et al., 2018). ART can improve the quality of the lives of patients with HIV and effectively reduce the spread of HIV by inhibiting its replication (Rodger et al., 2016). HIV Prevention Trials Network (HPTN) 052 programme trial’s interim result indicated that early ART prevented over 96% of HIV genetically transmission among serodiscordant couples (Cohen et al., 2016), while the final results of that trial demonstrated that early ART reduced 93% of HIV linkage than that was delayed ART (Cohen et al., 2020). A retrospective observational cohort study in China also exhibited a consistent result, compared with that late-ART initiation, the preventive efficacy of early-ART initiation among serodiscordant couples was 45%, and the preventive efficacy of mid-ART initiation was 39% (Liu et al., 2018). In the two phases of the People on ART-A New Evaluation of the Risks (PARTNER) (partners of PARTNER) study conducted in the European clinical base, ART and viral suppression proved to prevent sexual transmission among serodiscordant couples (Rodger et al., 2016, 2019). The HPTN 071 (PopART) trial, a large randomized population controlled clinical trial established at the community level conducted from 2013 through 2018, showed that ART and universal testing combination intervention can reduce HIV incidence and transmission at a population level (Hayes et al., 2019). There were reductions in HIV-associated mortality and HIV incidence when patients with HIV used ART.

Despite the goal of reaching 95-95-95 of the AIDS prevention and control strategy by 2030, very few studies currently exist on the transmission of HIV and critical factors at a population level globally. At present, most studies on HIV transmission are based on traditional epidemiological methods. Traditional epidemiological methods often require a large HIV cohort, long-term follow-up observation, and high attention. Furthermore, these need substantial financial budgets and human resources to support the follow-up efforts. In recent years, the development and application of genetic transmission network provide new ideas for the evaluation of HIV transmission (Oster et al., 2018). A genetic network refers to a group/s of people infected with HIV whose infectious viruses have a genetic similarity. HIV transmission network refers to the network formed by a group/s of people infected with HIV who have direct or indirect epidemiological links in the transmission process (National Center for HIV/AIDS, Viral Hepatitis, STD, TB Prevention, and Division of HIV/AIDS Prevention, 2018). In the HIV-1 genetic transmission network analysis based in San Mateo County, it was found that African Americans played an important role in community transmission (Dalai et al., 2018). A genetic network analysis in Guangzhou, China, revealed that undisclosed MSM plays an important role in HIV-1 transmission (Yan et al., 2020). A genomic and spatial epidemiological analysis identified high-risk populations and areas in Sichuan (Yuan et al., 2022). HIV transmission constructed by genetic transmission network analysis can determine the transmission population (Poon et al., 2016; de Oliveira et al., 2017; Dk et al., 2018) and also explore the characteristics and influencing factors of HIV transmission (Su et al., 2018; Wu et al., 2019; Kang et al., 2021). In our previous study, which focused on evaluating the effects of ART on HIV transmission, the longitudinal genetic network method was employed to build a genetic transmission network during 2015–2018 in the rural areas of Guangxi (Kang et al., 2021). However, this study had some limitations. First, this statistical method did not adjust factors, which may overestimate prevention efficacy in HIV transmission for ART, and longitudinal repeated measurement data were not well-utilized. Second, only the ART variable was considered, and this was unable to reveal epidemic characteristics of HIV transmission. Third, genetic linkages between newly diagnosed patients with HIV were not analyzed.

Guangxi Zhuang Autonomous Region (herein “Guangxi”) is located in Southwest China. By 2020, Guangxi had accounted for 9.3% of the total number of nationally reported HIV/AIDS cases while representing less than 4% of the national population and had the third highest number of HIV cases reported in China (Jia et al., 2022). Before 2006, the main HIV transmission route was through injected drug abuse. Thereafter, sexual transmission became the primary infection route (Chen et al., 2019a). Heterosexual intercourse became the dominant transmission route for HIV (99.0%), and HIV is now rapidly spreading among the elderly in rural areas of Guangxi (Chen et al., 2019b). Due to the variability of epidemic populations and patterns (Li et al., 2018; Chen et al., 2021), local HIV epidemic prevention and control programmes are challenging. Based on the constructed genetic transmission network from our previous study, genetic linkages between newly diagnosed patients with HIV at follow-up and baseline were calculated, which represents the HIV secondary generation transmission in this study. The generalized estimating equation (GEE) model was used to process longitudinal repeated measurement data. the epidemic characteristics of the HIV sexual transmission in Guangxi were investigated, and the role of factors, including ART for HIV transmission, was evaluated at a population level in the scale-up HIV testing and ART context.

## Materials and methods

### Study design and participants

Participants living in the Luzhai County and Liujiang District, which are two major HIV epidemic areas in Liuzhou Prefecture, were recruited. Liuzhou is located in the northeast of Central Guangxi. This study was a longitudinal genetic network analysis of patients with HIV in Guangxi from 2015 to 2018. Baseline data of patients with HIV were collected from those diagnosed in Guangxi who received care up until 31 December 2015, while follow-up data encompassed newly diagnosed patients with HIV from 2016 to 2018. Eligibility criteria were as follows: (1) at least 18 years old; (2) living with HIV; and (3) provided written informed consent. The study was approved by the institutional review committee of the Guangxi Center for Disease Control and Prevention.

### Data collection

The collected data included sociodemographic information and clinical information. Sociodemographic information included age, sex, ethnicity, education, marital status, occupation, route of HIV transmission, and county. Clinical information included year of HIV diagnoses, CD4 cell count before ART, year of ART initiation, and follow-up data such as follow-up time, VL at follow-up, and follow-up status (e.g., treatment, death, transferred, withdrawal, and loss to follow-up). The dropped out (withdrawal or loss to follow-up) was defined as withdrawal or loss to follow-up for more than 90 days. Individuals were followed up once every 3 months prior to receiving ART. The individuals were followed up at 0.5, 1, 2, and 3 months after receiving ART and then subsequently once every 3 months.

### Nucleic acid extraction amplification and sequencing

Whole blood samples were collected from patients with HIV. The plasma components were isolated and sent to the laboratory under cold chain conditions. DNA was extracted from 200 μl of whole blood using the QIAamp DNA Blood Mini Kit (Qiagen, Hilden, Germany), followed by the manufacturer’s protocol. RNA was extracted from 200 μl of plasma by using the QIAamp Viral RNA Mini Kit (Qiagen, Hilden, Germany). The HIV pol region was amplified by nested PCR using the extracted DNA and RNA as templates. The amplification and sequencing methods used in this study have been published previously (Liao et al., 2010; Xing et al., 2015).

### HIV-1 genetic transmission network

The HIV-1 genetic transmission network refers to a group/s of sequences that are not randomly gathered and have an epidemiological correlation. These sequences were obtained by sequencing and required a series of processes. They were spliced by BioEdit (Ibis Biosciences, Carlsbad, CA, United States; version 7.0.9.0) and aligned separately by the HIValign tool to obtain the final sequences used for analysis. The MEGA 7 software was used to identify the HIV-1 subtypes, and FastTree (developed by Morgan N. Price; version 1.3.1) constructed the phylogenetic tree. Before constructing the genetic transmission network, the distance between sequences (genetic distance [GD]) was used to represent the distance of patients with HIV. The pairwise Tamura-Nei (TN93) GD of the paired sequences was calculated using Hyphy (version 2.2.4). Finally, the HIV genetic transmission network was visualized by using the Cytoscape software. The genetic distance (GD) with the largest number of linked nodes and clusters was selected as the threshold for analyzing the genetic transmission network. In this study, 0.0075 was used as the GD threshold to calculate pairwise GD and identify linkage sequence pairs, as it had the most genetic networks and linkages.

The patients with early HIV symptoms were defined as those at baseline (e.g., December 31, 2015), and the corresponding newly diagnosed patients with HIV were defined as those diagnosed in the next year (e.g., those newly diagnosed in 2016). First, patients with HIV at baseline on December 31, 2015 were considered to construct genetic transmission network, and then the corresponding newly diagnosed patients with HIV in 2016 were put into the transmission network under the same GD. The genetic linkages between newly diagnosed patients with HIV in 2016 and those at baseline on December 31, 2015 were calculated. Second, based on the genetic transmission network constructed by patients with HIV at baseline on December 31, 2016, those newly diagnosed in 2017 were added into the network according to the same GD. Thereafter, the genetic transmission network between newly diagnosed patients with HIV in 2018 and those at baseline on December 31, 2017 was similarly constructed and the genetic linkages were similarly calculated.

### Statistical analysis

After constructing the three genetic transmission networks, the sociodemographic information and clinical information of the baseline characteristics of the three genetic transmission networks were observed repeatedly. Sociodemographic characteristics included age, sex, ethnicity, education, marital status, occupation, route of HIV transmission, and county. Clinical information included year of diagnosis, CD4 cell count before ART, and year of ART initiation. The genetic linkages between newly diagnosed patients with HIV during 2016–2018 and those at baseline during 2015–2017 were calculated. The number of genetic linkages was equal to the number of newly diagnosed patients with HIV linked to those at baseline.

The generalized estimating equation model was used to analyze the factors associated with HIV transmission through the genetic linkage between newly diagnosed patients with HIV during 2016–2018 and those at baseline during 2015–2017, respectively. GEE models could account for repeated and correlated measures within individuals and improve the statistical precision by using information across repeated measures (Ballinger, 2004). Taking the number of genetic linkages to present between newly diagnosed patients with HIV and those at baseline as the dependent variable, the unadjusted and adjusted odds ratio (AORs) were estimated for sociodemographic characteristics and clinical information in the GEE models. Furthermore, stratification analysis was performed to evaluate the impact of ART on the HIV transmission. Patients with HIV at each baseline were divided into treated group and untreated group, and the treated group was further stratified based on VL data available at baseline time point: treated HIV patients with VL of less than 50 copies/ml (ART with VL < 50) and at least 50 copies/ml. For ART without VL information, the follow-up status was used as the basis for further stratification. If withdrawal and loss to follow-up occurred in the follow-up status record, these patients with HIV were defined as treated HIV patients on dropout (ART on dropout); otherwise, patients with HIV were defined as treated HIV patients with VL missing (ART with VL missing). To control potential bias and avoid the influences of other variables, the adjusted baseline characteristics of age, sex, ethnicity, education, occupation, mobility, and route of HIV transmission were included as control variables in the multivariate generalized estimating equation model.

## Results

### Characteristics of patients with human immunodeficiency virus within the study

Of 3,259 identified HIV patient sequences, 2,714 patients were at baseline, and 545 were newly diagnosed patients with HIV at follow-up. The general characteristics of patients with HIV in this study are shown in Table 1. Overall, 51.0% of patients with HIV were 30–49 years old, and 36.0% were 50–69 years old. The majority of patients with HIV were men (61.8%). The proportion of HIV patients with Han ethnicity was 42.8%. More than half of patients with HIV (56.6%) had completed at least secondary education and 62.4% were married; among the married patients with HIV, 23.8% of the spouses of patients with HIV were HIV-positive, 13.6% of the spouses of those were HIV-negative, the remaining 24.8% of those lack spouse information, and 67.0% were farmers. The main route of HIV transmission was heterosexual intercourse (96.1%), and the main HIV-1 genetic subtype was CRF01_AE (93.4%). In addition, 80.2% of patients with HIV had CD4 cell counts of less than 350 cells/mm^3^ before receiving ART. Those lived in Luzhai County (62.5%) and Liujiang County (37.5%) in Guangxi of China.

**TABLE 1:** General characteristics of patients with HIV between 2015 and 2018 in Guangxi of China.

Variable	2015		2016		2017		2018		Total	
					
	N	%	N	%	N	%	N	%	N	%
Total	2,714	100.0	175	100.0	199	100.0	171	100.0	3,259	100.0
**Age, years**										
18∼29	214	7.9	13	7.4	16	8.0	14	8.2	257	7.9
30∼49	1,452	53.5	68	38.9	73	36.3	68	39.8	1,661	51.0
50∼69	914	33.7	87	49.7	93	46.7	81	47.4	1,175	36.0
≥70	134	4.9	7	4.0	17	8.5	8	4.7	166	5.1
**Sex**										
Male	1,629	60.0	120	68.6	143	71.9	123	71.9	2,015	61.8
Female	1,085	40.0	55	31.4	56	28.1	48	28.1	1,244	38.2
**Ethnicity**										
Han	1,231	45.4	56	32.0	60	30.2	48	28.1	1,395	42.8
Zhuang and other	1,483	54.7	119	68.0	139	69.8	123	71.9	1,864	57.2
**Education**										
No school or primary	1,131	41.7	95	54.3	114	57.3	76	44.4	1,416	43.5
Secondary school and above	1,583	58.3	80	45.7	85	42.6	95	55.5	1,843	56.6
**Marital status**										
Single	358	13.2	43	24.6	43	21.6	36	21.1	480	14.7
Married	1,756	64.7	88	50.3	110	55.3	80	46.8	2,034	62.4
Divorced/widowed	599	22.1	42	24.0	41	20.6	46	26.9	728	22.3
Unknown	1	0.0	2	1.1	5	2.5	9	5.3	17	0.5
**Occupation**										
Farmer	1,797	66.2	115	65.7	144	72.4	128	74.9	2,184	67.0
Other	917	33.8	60	34.3	55	27.6	43	25.2	1,075	33.0
**Route of HIV transmission**										
Heterosexual intercourse	2,654	97.8	159	90.9	170	85.4	149	87.1	3,132	96.1
Homosexual intercourse	18	0.7	4	2.3	3	1.5	1	0.6	26	0.8
Unknown	42	1.5	12	6.9	26	13.1	21	12.3	101	3.1
**Genetic subtype**										
CRF01_AE	2,576	94.9	153	87.4	170	85.5	148	86.5	3,047	93.4
CRF07_BC	71	2.6	8	4.6	10	5.0	11	6.4	100	3.1
CRF08_BC	39	1.4	5	2.9	14	7.0	7	4.1	65	2.0
Other	28	1.0	9	5.1	5	2.5	5	3.0	47	1.4
**CD4 count before ART**										
<350 cells/mm^3^	2,284	84.2	118	67.4	109	54.8	103	60.2	2,614	80.2
≥350 cells/mm^3^	287	10.6	17	9.7	17	8.5	19	11.1	340	10.4
Missing	143	5.3	40	22.9	73	36.7	49	28.7	305	9.4
**County**										
Luzhai	1,794	66.1	98	56.0	69	34.7	67	39.2	2,038	62.5
Liujiang	910	33.5	77	44.0	130	65.3	104	60.8	1,221	37.5
**Viral load after 12 months of ART**										
<50 copies/mL	814	30.0	61	34.9	43	21.6	1	0.6	919	28.2
≥50 copies/mL	134	4.9	7	4.0	16	8.0	0	0.0	157	4.8
Missing*	1655	61.0	67	38.3	68	34.2	121	70.8	1911	58.6

*Including viral load information of HIV patients with ART time less than 6 months.

### HIV-1 genetic transmission network

**FIGURE 1:**
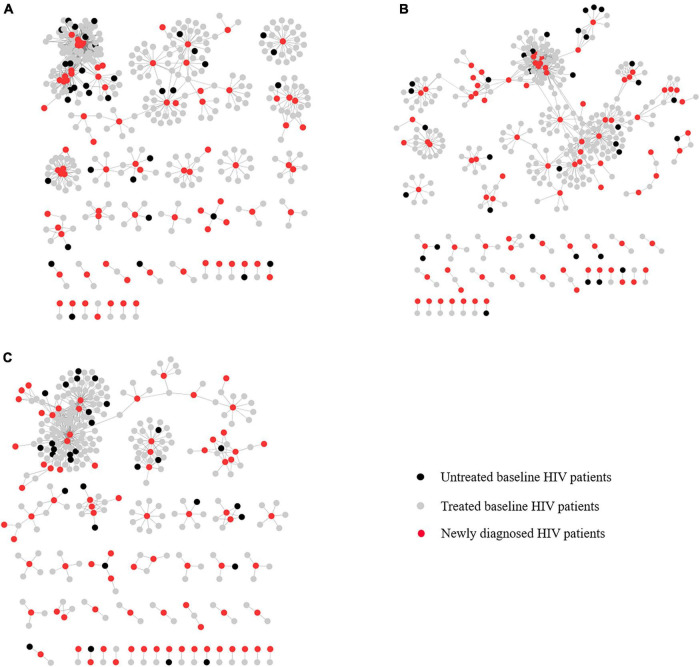
Transmission network of genetic linkage between newly diagnosed patients with HIV and those at baseline in Guangxi of China. **(A)** Genetic linkage between newly diagnosed patients with HIV in 2016 and those at baseline in 2015. **(B)** Genetic linkage between newly diagnosed patients with HIV in 2017 and those at baseline in 2016. **(C)** Genetic linkage between newly diagnosed patients with HIV in 2018 and those at baseline in 2017.

The genetic transmission networks between newly diagnosed patients with HIV during 2016–2018 and those at baseline during 2015–2017 are shown in Figure 1. There were a total of 8,691 baseline observations in the generalized estimating equation model. Among the patients with HIV at baseline within the networks, 466 and 384 were linked to one newly diagnosed patient with HIV and more than two newly diagnosed HIV patients with a GD threshold of 0.0075, respectively (Table 2). Approximately, 14.1% (98/694) of patients with HIV at baseline did not receive ART linked to the those newly diagnosed. About 13.9% (417/3,009) of patients with HIV at baseline aged 50–60 years were linked to those newly diagnosed. The proportion of patients with HIV at baseline aged more than 70 years linked to those newly diagnosed was 20.1% (87/433). Approximately, 11.8% (621/5,270) of male patients with HIV at baseline were linked to those newly diagnosed. The proportion of Zhuang and other nationalities linked to newly diagnosed patients with HIV was 12.0% (577/4,826). About 12.8% (473/3,697) of patients with HIV at baseline with primary schooling as their highest level of educational attainment were linked to those newly diagnosed. The proportion of married patients with HIV at baseline linked to those newly diagnosed was 10.2% (567/5,554). Approximately, 11.4% (659/5,765) of patients with HIV at baseline who were farmers were linked to those newly diagnosed. The proportion of patients with HIV at baseline who were infected with HIV through heterosexual intercourse route linked to those newly diagnosed was 10.0% (841/8,450). Finally, the proportions of patients with HIV at baseline with CRF01_AE subtype, CD4 cell counts above 350 cells/mm^3^, and living in Liujiang linked to those newly diagnosed were 10.1 (829/8,204), 12.1 (110/912), and 15.8% (476/3,014), respectively.

**TABLE 2:** Genetic linkage between newly diagnosed patients with HIV during 2016–2018 and those at baseline during 2015–2017 in Guangxi of China, respectively.

Variable	Baseline	Number of genetic linkages between newly diagnosed HIV patients and HIV patients at baseline			*OR* (95% CI)	*P*	*AOR* (95% CI)	*P*
		
		1 person (%)	≥2 person (%)	≥1 person (%)				
Total	8691	466 (5.4)	384 (4.4)	850 (9.8)				
**Age, year**								
18∼29	684	18 (2.6)	9 (1.3)	27 (3.9)	1.00		1.00	
30∼49	4565	191 (4.2)	128 (2.8)	319 (7.0)	1.83 (1.08–3.10)	0.024	1.75 (1.03–2.98)	0.039
50∼69	3009	210 (7.0)	207 (6.9)	417 (13.9)	3.95 (2.34–6.68)	<0.001	3.72 (2.16–6.39)	<0.001
≥70	433	47 (10.9)	40 (9.2)	87 (20.1)	6.12 (3.37–11.12)	<0.001	5.12 (2.77–9.46)	<0.001
**Sex**								
Female	3421	137 (4.0)	92 (2.7)	229 (6.7)	1.00		1.00	
Male	5270	329 (6.2)	292 (5.5)	621 (11.8)	1.87 (1.51–2.32)	<0.001	1.82 (1.46–2.28)	<0.001
**Ethnicity**								
Han	3865	177 (4.6)	96 (2.5)	273 (7.1)	1.00		1.00	
Zhuang and other	4826	289 (6.0)	288 (6.0)	577 (12.0)	1.81 (1.48–2.22)	<0.001	1.50 (1.22–1.84)	<0.001
**Education**								
Secondary school and above	4994	213 (4.3)	164 (3.3)	377 (7.5)	1.00		1.00	
Illiteracy or primary	3697	253 (6.8)	220 (6.0)	473 (12.8)	1.80 (1.48–2.19)	<0.001	1.43 (1.15–1.77)	<0.001
**Marital status**								
Single	1203	54 (4.5)	42 (3.5)	96 (8.0)	1.00			
Married	5554	307 (5.5)	260 (4.7)	567 (10.2)	1.31 (0.96–1.79)	0.085		
Divorced/widowed	1922	102 (5.3)	82 (4.3)	184 (9.6)	1.22 (0.86–1.73)	0.264		
Unknown	12	3 (25.0)	0 (0.0)	3 (25.0)	3.31 (0.90–12.22)	0.073		
**Occupation**								
Other	2926	113 (3.9)	78 (2.7)	191 (6.5)	1.00		1.00	
Farmer	5765	353 (6.1)	306 (5.3)	659 (11.4)	1.85 (1.48–2.32)	<0.001	1.48 (1.17–1.87)	0.001
**Route of HIV transmission**								
Other or unknown	241	6 (2.5)	3 (1.2)	9 (3.7)	1.00		1.00	
Heterosexual intercourse	8450	460 (5.4)	381 (4.5)	841 (10.0)	2.86 (1.39–5.88)	0.004	3.19 (1.42–7.16)	0.005
**Antiretroviral therapy**								
Untreated	694	50 (7.2)	48 (6.9)	98 (14.1)	1.00		1.00	
Treated	7997	416 (5.2)	336 (4.2)	752 (9.4)	0.63 (0.46–0.85)	0.003	0.67 (0.48–0.93)	0.016
**Genetic subtype**								
CRF01_AE	8204	451 (5.5)	378 (4.6)	829 (10.1)	1.00			
Other	487	15 (3.1)	6 (1.2)	21 (4.3)	0.40 (0.25–0.64)	<0.001		
**CD4 count before ART, cells/mm^3^**								
<350	7197	373 (5.2)	289 (4.0)	662 (9.2)	1.00			
≥350	912	55 (6.0)	55 (6.0)	110 (12.1)	1.36 (1.01–1.85)	0.045		
Missing	582	38 (6.5)	40 (6.9)	78 (13.4)	1.54 (1.08–2.20)	0.017		
**County**								
Luzhai	5677	266 (4.7)	108 (1.9)	374 (6.6)	1.00			
Liujiang	3014	200 (6.6)	276 (9.2)	476 (15.8)	2.75 (2.26–3.34)	<0.001		
**Mobility**								
Not migrated	4742	226 (4.8)	81 (1.7)	307 (6.5)	1.00			
Migrated	3949	240 (6.1)	303 (7.7)	543 (13.8)	2.37 (1.95–2.87)	<0.001	2.72 (2.21–3.34)	<0.001

To determine the factors associated with genetic linkage, the generalized estimating equation model was conducted. According to the univariate generalized estimating equation model, age, sex, ethnicity, education, occupation, route of HIV transmission, ART, genetic subtype, CD4 count before ART, and county factors were significantly associated with HIV transmission through the genetic network between newly diagnosed patients with HIV during 2016–2018 and those at baseline during 2015–2017, respectively. After adjustments in the final generalized estimating equation model, treated HIV patients were significantly associated with less HIV transmission through the genetic network (AOR: 0.67, 95% CI: 0.48–0.93, *P* = 0.016). In contrast, other factors significantly associated with HIV transmission through the genetic network included being aged 50–69 years (AOR: 3.72, 95% CI: 2.16–6.39; reference group: 18–29 years old, *P* < 0.001), over 70 years old (AOR: 5.12, 95% CI: 2.77–9.46; reference group: 18–29 years old, *P* < 0.001), men (AOR: 1.82, 95% CI: 1.46–2.28, *P* < 0.001), Zhuang and other nationalities (AOR: 1.50, 95% CI: 1.22–1.84; reference group: Han, *P* < 0.001), illiteracy or only having primary school educational attainment (AOR: 1.43, 95% CI: 1.15–1.77; reference group: secondary schooling and above, *P* < 0.001), working as a farmer (AOR: 1.48, 95% CI: 1.17–1.87; reference group: others, *P* = 0.001), heterosexual intercourse route (AOR: 3.19, 95% CI: 1.42–7.16; reference group: other or unknown, *P* = 0.005), and migrated (AOR: 2.72, 95% CI: 2.21–3.34; reference group: not migrated, *P* < 0.001).

### Genetic linkage between newly diagnosed patients with HIV and those at baseline, stratified by treatment, viral load, and dropout

**FIGURE 2:**
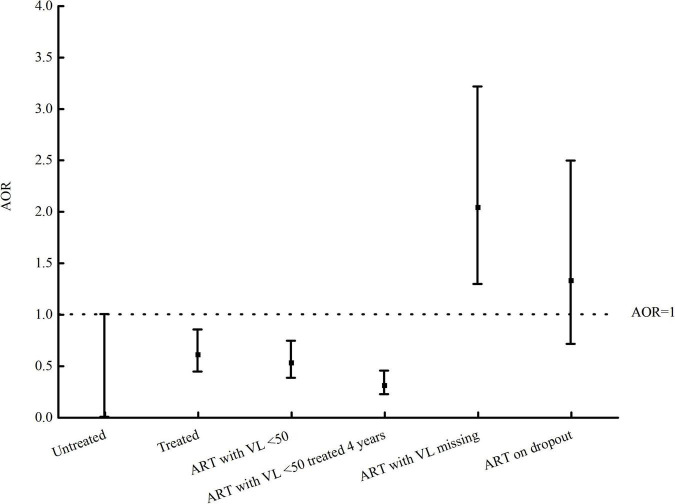
AOR values of stratified analysis of genetic transmission network in Guangxi of China.

The treated HIV patients at baseline were divided into subgroups by treatment, VL, and dropout (Figure 2 and Table 3). The proportion of treated HIV patients at baseline with a VL of less than 50 copies/ml linked to those newly diagnosed was 8.2% (583/7,088). Only 4.9% (214/4,395) of patients with HIV who were treated for 4 years and had a VL of less than 50 copies/ml at baseline were linked to those newly diagnosed at baseline. The proportion of treated HIV patients with VL missing at baseline and treated HIV patients on dropout at baseline genetically linked to those newly diagnosed was 27.8 (66/237) and 20.4% (33/162), respectively. The median (IQR) ART duration in treated HIV patients with VL missing was 269 days (192–567). Compared with untreated HIV patients, treated HIV patients with VL of less than 50 copies/ml were associated with less likelihood of HIV transmission (AOR: 0.59, 95% CI: 0.43–0.82; *P* = 0.002). Patients with HIV who were treated for 4 years and had a VL of less than 50 copies/ml at baseline were associated with less likelihood of HIV transmission (AOR: 0.35, 95% CI: 0.24–0.50; *P* < 0.001). Treated HIV patients with VL missing at baseline were associated with HIV transmission (AOR: 1.64, 95% CI: 1.03–2.60; *P* = 0.038). There was no significant difference in the proportion of genetic linkages between untreated HIV patients at baseline and treated HIV patients on dropout at baseline.

**TABLE 3:** Genetic linkage between newly diagnosed patients with HIV and those at baseline in Guangxi of China, stratified by treatment, viral load, and follow-up status.

Variable	Baseline	Number of genetic linkages between newly diagnosed HIV patients and HIV patients at baseline			*OR* (95% CI)	*P*	*AOR* (95% CI)	*P*
		
		1 person (%)	≥2 person (%)	≥1 person (%)				
Total	8691	466 (5.4)	384 (4.4)	850 (9.8)				
**Model 1**								
Untreated	694	50 (7.2)	48 (6.9)	98 (14.1)	1.00		1.00	
Treated	7997	416 (5.2)	336 (4.2)	752 (9.4)	0.63 (0.46–0.85)	0.003	0.67 (0.48–0.93)	0.016
Treated HIV patients with VL < 50 copies/mL	7088	337 (4.8)	246 (3.5)	583 (8.2)	0.54 (0.40–0.74)	<0.001	0.59 (0.43–0.82)	0.002
**Model 2**								
Untreated	694	50 (7.2)	48 (6.9)	98 (14.1)	1.00		1.00	
Treated	7997	416 (5.2)	336 (4.2)	752 (9.4)	0.63 (0.46–0.85)	0.003	0.67 (0.48–0.93)	0.016
Treated HIV patients with VL < 50 copies/mL and treated more than 4 years	4395	155 (3.5)	59 (1.3)	214 (4.9)	0.31 (0.22–0.43)	<0.001	0.35 (0.24–0.50)	<0.001
**Model 3**								
Untreated	694	50 (7.2)	48 (6.9)	98 (14.1)	1.00		1.00	
Treated	7997	416 (5.2)	336 (4.2)	752 (9.4)	0.63 (0.46–0.85)	0.003	0.67 (0.48–0.93)	0.016
Treated HIV patients with VL missing	237	25 (10.5)	41 (17.3)	66 (27.8)	2.44 (1.59–3.76)	<0.001	1.64 (1.03–2.60)	0.038
Treated HIV patients on dropout	162	12 (7.4)	21 (13.0)	33 (20.4)	1.61 (0.86–3.01)	0.133	1.15 (0.61–2.16)	0.674

Model 1: Stratified by VL; Model 2: Stratified by VL and treatment; Model 3: Stratified by VL and follow-up status.

### Sensitivity analysis

To avoid the late HIV diagnosis phenomenon, newly infected patients with HIV, diagnosed from 2016 to 2018, with a CD4 count of more than 200 cells/mm^3^ at the first visit, were included in this study. Treated patients were significantly associated with less HIV transmission through the genetic network (AOR: 0.62, 95% CI: 0.41–0.95, *P* = 0.029) in the final GEE model.

Patients with HIV undergoing their first infection year are more likely to transmit HIV (Ratmann et al., 2016). Based on linkages between newly diagnosed and baseline patients with HIV, the genetic linkages between newly diagnosed patients themselves were analyzed. Treated patients were significantly associated with less HIV transmission through the genetic network (AOR: 0.65, 95% CI: 0.52–0.82, *P* < 0.001) in the final GEE model.

According to the National Technical Guideline for HIV Transmission Network Monitoring and Intervention in China, the distance threshold (0.0075) resulting in the maximum number of clusters was used as the final GD threshold. The sensitivity of the GD was also analyzed. Furthermore, the prevention efficacy in HIV transmission for patients with HIV was similar when the GD threshold was 0.0065 and 0.0085, respectively (Supplementary Table 1).

## Discussion

Our longitudinal genetic network study in Guangxi from 2015 to 2018 indicated that ART reduced the risk of HIV transmission by 33% at a population level. Previous studies mainly using traditional epidemiological methods have indicated that ART can reduce the risk of HIV transmission among serodiscordant couples (Eshleman et al., 2017; Ma et al., 2019). The HPTN 071 clinic trial also confirmed the effect of ART in reducing HIV transmission at the population level (Hayes et al., 2019). The previous study, which did not consider the epidemiological factors, has only shown that ART reduced HIV transmission by 53.6% (Kang et al., 2021). Compared with our results in this study, we adjusted the age, sex, ethnicity, education, occupation, mobility, and route of HIV transmission variables, which was more accurately evaluated. In the context of ART scale-up, our study is the first to evaluate the effect of ART on the prevention of HIV transmission at the population level in real-world settings through a longitudinal genetic network analysis. The stratification results showed that the prevention efficacy in HIV transmission for treated HIV patients with a VL of less than 50 copies/ml was 41%. Other studies demonstrated that the virus has been inhibited after ART, and HIV transmission risk can be greatly reduced (Bavinton et al., 2018; LeMessurier et al., 2018). The increased risk of HIV transmission was related to the high VL of patients with HIV (Eshleman et al., 2017; Liu et al., 2018; Eisinger et al., 2019). Our study results also showed that the prevention efficacy in HIV transmission for patients with HIV treated for 4 years and with a VL of less than 50 copies/ml was 65%. The GD threshold of 0.0075 showed that the evolution time of the HIV strain is 4–5 years (National Center for HIV/AIDS, Viral Hepatitis, STD, TB Prevention, and Division of HIV/AIDS Prevention, 2018). Therefore, the time of receiving ART for more than 4 years can abandon irrelevant genetic linkages when building a genetic transmission network, which can show the true effect of ART in preventing HIV transmission. Our study results provide strong evidence to support the World Health Organization’s recommendation for treat-all prevention at the population level.

Our study determined that there was no significant reduction in HIV transmission among treated HIV patients with VL missing or those on dropout. Our previous study also found that VL testing was used as a proxy for treatment adherence that was significantly associated with virological failure and death (Shen et al., 2016; Zhang et al., 2021). If patients with HIV stop receiving ART, VL may rebound to a level associated with an increased risk of HIV transmission, which will also increase the drug resistance rate of those (Lei et al., 2018). In our previous study, we demonstrated that a high proportion of patients with HIV receiving ART were dropped out in China (Tang et al., 2017; Kang et al., 2019), and the overall attrition rate was 10.86 per 100 person-years in HIV-infected patients who started ART between 2012 and 2015 (Zhu et al., 2021). This study result demonstrates that more attention should be given to ART management and care should be paid on improving the drug adherence of patients with HIV, strengthening the service quality of ART clinics, and conducting VL testing promptly as required. Under the context of ART scale-up, VL monitoring for patients with HIV who received ART can effectively prevent the HIV transmission and reduce death, which provides support for the achievement of the “95-95-95” goal by 2030.

Our study found that migration was significantly associated with HIV transmission, and there are few studies evaluating the impact of migrant population on HIV transmission among heterosexual intercourse at a population level in China. Our study also found that individuals older than 50 years, male, Zhuang and other nationalities, with less than secondary schooling, working as farmers, and heterosexual transmission were at a higher risk of HIV transmission. Older men with low education had a high risk of HIV heterosexual transmission (Wu et al., 2019). By the end of 2021, the proportion of older patients with HIV (more than 50 years of age) reported to be living with HIV/AIDS was 61.4 and 45.7% in Guangxi and China, respectively. HIV is already spreading beyond traditional high-risk individuals, such as injection drug users, female sex workers, and men who have sex with men, to populations that include older heterosexual men in rural areas of China. These new changes in HIV transmission patterns pose greater challenges for new prevention efforts focused on older individuals within the general population. In addition to providing a treat-all prevention strategy, targeted precision prevention and interventions, such as through safe sex education (condom use of sex), HIV testing and counseling, immediate ART, and improve ART adherence, are urgently needed for addressing heterosexual HIV transmission among the older men within the general population.

This study had some limitations. First, a GD of 0.0075 was chosen, which represents the evolution of HIV strains and transmission in 4–5 years. In China, the prevalence of late HIV diagnosis has reached 43% (Sun et al., 2021). We do not have data on those newly infected patients with HIV, which may lead to a reduction in statistical power for the current analysis. Second, the sample size of stratified analysis groups such as treated HIV patients with VL missing may be not large enough. Finally, this study did not adjust the risk tolerance. It seems possible that individuals who do not pay attention to medical care or have a greater tolerance for risk may have a risk of sexual intercourse and have also not been receiving ART.

## Conclusion

Understanding the networks of sexually transmitted HIV infections in rural Southwest China at a population level during the expansion of ART is critical. This study reveals the role of ART in reducing HIV transmission in the real world. Ending the HIV epidemic requires a series of comprehensive measures, such as patients with HIV receiving ART immediately after diagnosis, treatment improvements, and focused precision intervention on high-risk individuals during the expansion of ART.

## Data availability statement

The data analyzed in this study is subject to the following licenses/restrictions: data not publicly available but could be obtained upon request and approval from Chinese Center for Disease Control and Prevention (China CDC). Requests to access these datasets should be directed to YR, ruanyuhua92@chinaaids.cn.

## Ethics statement

The studies involving human participants were reviewed and approved by National Center for AIDS/STD Control and Prevention, Chinese Center for Disease Control and Prevention, China. The patients/participants provided their written informed consent to participate in this study.

## Author contributions

JC, LJL, YF, and YR were responsible for study design and planning. HC, JL, LHL, ZS, JZ, HL, GL, QZ, and SL contributed to data collection and management. JC, RK, and YR contributed to data analysis. JC, HX, LJL, YS, and YR contributed to interpretation. JC and YR contributed to writing the report. All authors read and approved the final version of the article.
