# Supplementary material for: Genetic network analysis of human immunodeficiency virus sexual transmission in rural Southwest China after the expansion of antiretroviral therapy: A population-based study
Source: Front Microbiol. 2022 Aug 18;13:962477. doi: 10.3389/fmicb.2022.962477 (PMC9434148; doi:10.3389/fmicb.2022.962477)
Supplement: Supplementary file 1 [file Table_1.docx]

Supplementary Material

**Supplemental table 1. Sensitivity analysis of genetic linkage between newly diagnosed HIV patients with untreated and treated HIV patients at baseline in Guangxi of China, stratified by genetic distance, respectively**

| Variable | Baseline | Number of genetic linkages between newly diagnosed HIV patients and HIV patients at baseline | | | *OR (95%CI)* | *P* | *AOR (95%CI)* | *P* |
| --- | --- | --- | --- | --- | --- | --- | --- | --- |
|  |  | 1 person (%) | ≥2 person (%) | ≥1 person (%) |  |  |  |  |
| 0.65% genetic threshold |  |  |  |  |  |  |  |  |
| Total | 8691 | 369 (4.2) | 259 (3.0) | 628 (7.2) |  |  |  |  |
| Untreated | 694 | 44 (6.3) | 38 (5.5) | 82 (11.8) | 1.00 |  | 1.00 |  |
| Treated | 7997 | 325 (4.1) | 221 (2.8) | 546 (6.8) | 0.54 (0.39-0.75) | <.001 | 0.58 (0.41-0.82) | 0.0019 |
| 0.75% genetic threshold |  |  |  |  |  |  |  |  |
| Total | 8691 | 466 (5.4) | 384 (4.4) | 850 (9.8) |  |  |  |  |
| Untreated | 694 | 50 (7.2) | 48 (6.9) | 98 (14.1) | 1.00 |  | 1.00 |  |
| Treated | 7997 | 416 (5.2) | 336 (4.2) | 752 (9.4) | 0.63 (0.46-0.85) | 0.003 | 0.67 (0.48-0.93) | 0.016 |
| 0.85% genetic threshold |  |  |  |  |  |  |  |  |
| Total | 8691 | 617 (7.1) | 536 (6.2) | 1153 (13.3) |  |  |  |  |
| Untreated | 694 | 71 (10.2) | 57 (8.2) | 128 (18.4) | 1.00 |  | 1.00 |  |
| Treated | 7997 | 546 (6.8) | 479 (6.0) | 1025 (12.8) | 0.66 (0.50-0.85) | 0.002 | 0.68 (0.51-0.91) | 0.009 |

**Supplemental table 2. Genetic linkage between newly diagnosed HIV patients with HIV patients at baseline in Guangxi of China,** **stratified by treatment and viral load**

| Variable | Baseline | Number of genetic linkages between newly diagnosed HIV patients and HIV patients at baseline | | | *OR (95%CI)* | *P* | *AOR (95%CI)* | *P* |
| --- | --- | --- | --- | --- | --- | --- | --- | --- |
|  |  | 1 person (%) | ≥2 person (%) | ≥1 person (%) |  |  |  |  |
| Total | 8691 | 466 (5.4) | 384 (4.4) | 850 (9.8) |  |  |  |  |
| Untreated | 694 | 50 (7.2) | 48 (6.9) | 98 (14.1) | 1.00 |  | 1.00 |  |
| Treated | 7997 | 416 (5.2) | 336 (4.2) | 752 (9.4) | 0.63 (0.46-0.85) | 0.003 | 0.67 (0.48-0.93) | 0.016 |
| Treated HIV patients with VL ≥50 copies/mL | 510 | 42 (8.2) | 28 (5.5) | 70 (13.7) | 0.95 (0.62-1.46) | 0.827 | 0.88 (0.57-1.37) | 0.574 |
